# Supplementary material for: Lignicolous Freshwater Fungi from Plateau Lakes in China (I): Morphological and Phylogenetic Analyses Reveal Eight Species of Lentitheciaceae, Including New Genus, New Species and New Records
Source: J Fungi (Basel). 2023 Sep 25;9(10):962. doi: 10.3390/jof9100962 (PMC10607872; doi:10.3390/jof9100962)
Supplement: Supplementary file 1 [file jof-09-00962-s001.zip › Figure S1.pdf]

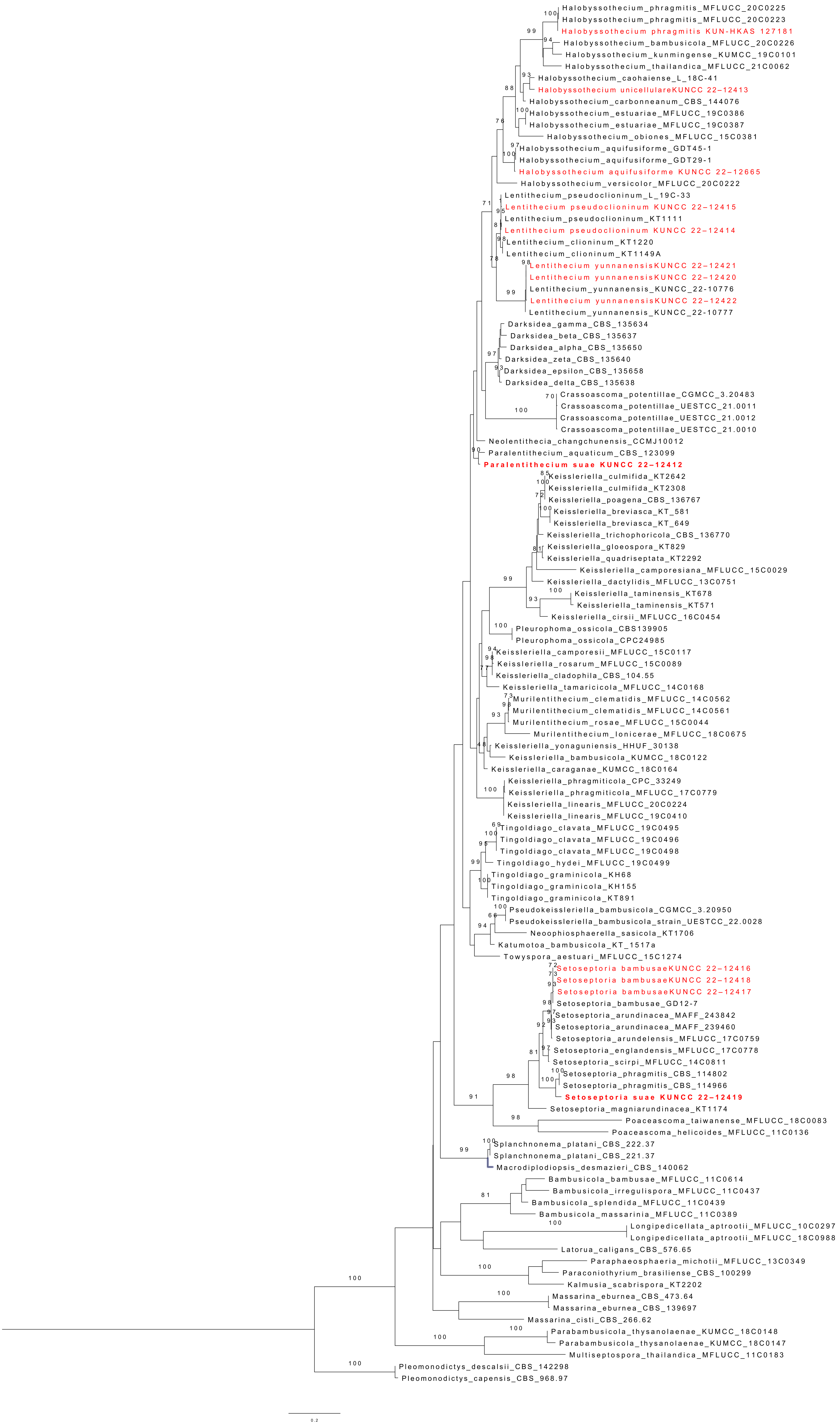

Figure S1. ML phylogenetic tree of Lentitheciaceae inferred from the ITS sequences (539 bp). Support in nodes is indicated above by bootstrap values of more than 70 %. New species are indicated in red bold. New strains are indicated in red.
